# Rapid maxillary expansion effects: An alternative assessment method by means of cone-beam tomography

**DOI:** 10.1590/2176-9451.19.5.088-096.oar

**Published:** 2014

**Authors:** Camilo Aquino Melgaço, José Columbano, Estela Maris Jurach, Matilde da Cunha Gonçalves Nojima, Eduardo Franzotti Sant'Anna, Lincoln Issamu Nojima

**Affiliations:** 1 Professor, Rio Verde Valley University (UNINCOR); 2 Assistant professor, São José College; 3 Assistant professor, Federal University of Santa Maria (UFSM); 4 Adjunct professor, Federal University of Rio de Janeiro (UFRJ)

**Keywords:** Orthodontics, Malocclusion, Palatal expander

## Abstract

**INTRODUCTION:**

This study aims to develop a method to assess the changes in palatal and lingual cross-sectional areas in patients submitted to rapid maxillary expansion (RME).

**METHODS:**

The sample comprised 31 Class I malocclusion individuals submitted to RME and divided into two groups treated with Haas (17 patients) and Hyrax (14 patients) expanders. Cone-beam computed tomography scans were acquired at T_0_ (before expansion ) and T_1_ (six months after screw stabilization). Maxillary and mandibular cross-sectional areas were assessed at first permanent molars and first premolars regions and compared at T_0_ and T_1_. Mandibular occlusal area was also analyzed.

**RESULTS:**

Maxillary cross-sectional areas increased in 56.18 mm^2^ and 44.32 mm^2^ for the posterior and anterior regions. These values were smaller for the mandible, representing augmentation of 40.32 mm^2^ and 39.91 mm^2^ for posterior and anterior sections. No differences were found when comparing both expanders. Mandibular occlusal area increased 43.99mm^2^ and mandibular incisors proclined. Increments of 1.74 mm and 1.7 mm occurred in mandibular intermolar and interpremolar distances. These same distances presented increments of 5.5 mm and 5.57 mm for the maxillary arch.

**CONCLUSION:**

Occlusal and cross-sectional areas increased significantly after RME. The method described seems to be reliable and precise to assess intraoral area changes.

## Abstract (pt)

**INTRODUÇÃO:**

o presente estudo teve como objetivo desenvolver um método para avaliar as mudanças nas áreas transversais palatinas e linguais em pacientes submetidos à expansão rápida da maxila (ERM).

**MÉTODOS:**

a amostra foi composta por 31 indivíduos com má oclusão Classe I de Angle, submetidos a ERM e divididos em dois grupos, tratados com expansores tipo Haas (17 pacientes) e de Hyrax (14 pacientes). Tomografias computadorizadas de feixe cônico foram adquiridas em T_0_ e T_1_ (antes da expansão e seis meses após a estabilização do parafuso). Áreas transversais da maxila e mandíbula foram avaliadas nas regiões de primeiros molares permanentes e pré-molares e comparadas entre T_0_ e T_1_. A área oclusal mandibular também foi analisada.

**RESULTADOS:**

as áreas transversais maxilares aumentaram 56,18mm^2^ e 44,32mm^2^ para regiões posterior e anterior, respectivamente. Esses valores foram menores para mandíbula, representando aumentos de 40,32mm^2^ e de 39,91mm^2^ para as seções anterior e posterior. Não foram encontradas diferenças quando se comparam os dois expansores. A área oclusal mandibular aumentou 43,99mm^2^ e incisivos inferiores vestibularizaram. Incrementos de 1,74mm e 1,7mm ocorreram entre as distâncias intermolares e interpré-molares inferiores. Essas mesmas distâncias apresentaram incrementos de 5,5mm e de 5,57mm para maxila.

**CONCLUSÃO:**

as áreas transversais avaliadas e oclusal de mandíbula aumentaram significativamente após a ERM. O processo descrito parece ser um método confiável e preciso para avaliar as mudanças das área intrabucais propostas.

## INTRODUCTION

Midpalatal suture in the maxilla might be split by rapid maxillary expansion (RME), a method first described in 1860.^2^ Many studies based on linear and angular analyses confirm the dentoalveolar and skeletal changes induced by this procedure.^10^^,^ 11 ^,^ 24 ^,^ 34 Increased maxillary transverse dimension is key not only to achieve space gain for teeth alignment, but also to improve stomatognathic functions, such as nasal cavity enlargement, and favor better tongue position.^1^^,^ 11 ^,^ 12 ^,^ 13 ^,^ 24 When compared to normal arches, patients with maxillary constriction have their tongue in a lower position.^11^ Expansion of mandibular arch widths is also observed after RME.^1^^,^ 11 ^,^ 13 In these cases, altered dental contacts could incline posterior mandibular teeth buccally.^10^^,^ 13 ^,^ 15 ^,^ 16 Long-term outcomes indicate spontaneous mandibular arch response in Class I malocclusion patients treated with RME only, thereby showing clinical stability and significant augmentation of mandibular intermolar and intercanine widths.^24^^,^ 27 ^,^ 28 ^,^ 32

Two types of expanders are most commonly used. Because the tooth-tissue borne expander (Haas) has an acrylic pad in contact with the palate, it distributes expanding forces along posterior teeth and the palatal vault. Conversely, the tooth-borne expander (Hyrax) does not have this acrylic pad and, for this reason, it only presumably delivers forces to the maxilla by means of appliance-supporting teeth.^6^ Some authors reported similar effects for both Haas and Hyrax expanders; however, other studies suggest less teeth inclination when tooth-tissue borne expander is used.^6^^,^ 7 ^,^ 14 ^,^ 29 ^,^ 30 ^,^ 35 Nowadays, highly developed techniques based on tomographic images and 3D models are available and used to assess morphological changes of the dentofacial complex.^9^^,^ 20 ^,^ 30 ^,^ 33 However, the impact of RME treatment on intraoral space gain has not been fully explored.^30^^,^ 33

This study aimed at developing a method to assess palatal and lingual cross-sectional changes in Class I malocclusion patients submitted to RME.

## MATERIAL AND METHODS

A total of 467 adolescents from five high schools of Belo Horizonte/Brazil were examined for potential RME treatment. In selecting the sample, the following inclusion criteria were applied: Angle Class I malocclusion; clinical need for rapid maxillary expansion visually determined by excessive palatal crown inclination of posterior maxillary teeth; no posterior or anterior crossbite; good oral health conditions (no periodontal disease or tooth decay); clinically healthy temporomandibular joints with normal range of motion; and no functional deviations. The exclusion criteria were: Congenitally missing teeth; craniofacial deformity; systemic diseases; history or evidence of disk displacement, pain or joint noises. Permanent dentition without previous orthodontic treatment was required for both arches (except for third molars).

Initial sample comprised 58 individuals; however, only 34 patients with average age of 12 years and 10 months for girls and 13 years for boys, with active facial growth (posteriorly confirmed by cervical vertebral maturation method^24^), started the treatment. The sample was randomly and equally divided into Group I (Haas) and Group II (Hyrax). Cone-beam computed tomography (CBCT) was taken before adaptation of expanders (T_0_) and 6 months after screw stabilization (T_1_). During the retention period, three patients were eliminated from the study due to premature appliance removal; therefore, 17 patients remained in Group I while 14 patients remained in Group II with a total sample comprising 31 individuals. This project was approved by the Federal University of Rio de Janeiro Institutional Review Board (n^o^. 35/2010 process n^o^. 62/2009). An informed consent form was signed by all patients' parents or guardians.

The same laboratory manufactured all appliances using 11-mm screws (Dental Morelli, São Paulo, Brazil). All first premolars and first molars were banded and received 1.0-mm stainless steel wires welded to the palatal and buccal surfaces of bands. During the first activation phase, the screws were opened 0.8 mm (a complete turn). Subsequently, activations were based on the same protocol adopted by several authors^12^^,^ 18 ^,^ 25 ^,^ 31 and consisted of a quarter of a turn (0.2 mm) in the morning and a quarter of a turn in the evening. The screws were stabilized when the tip of the palatal cusps of the maxillary permanent first molars contacted the tip of buccal cusps of the mandibular permanent first molars, as determined by clinical observation.

During CBCT scanning, all patients were oriented to remain in maximal dental intercuspation with their heads positioned so that the Frankfort and mid-sagittal planes were oriented parallel and perpendicular to the floor, respectively. The same equipment (i-CAT, Imaging Sciences International, Hatfield, PA, USA) was used according to a standard protocol (120 KVp, 5 mA, FOV = 13 x 17, voxel = 0.4 mm and scan time = 20 sec). Data were exported in DICOM (Digital Imaging and Communication in Medicine) format and imported into Dolphin Imaging software^(r)^ (version 11.0 - Dolphin Imaging & Management Solutions, Charsworth, CA, USA) so as to reconstruct 3D images for further analysis.

After images were obtained, the following landmarks were established: Maxilla - tip of first premolars palatal cusps and first permanent molars mesio-palatal cusps. Mandible - the center mesio-distal width of the incisal border of four incisors, tips of both canines, buccal cusps of all premolars, tips of first permanent molars mesio-buccal and middle cusps.

**Figure 1:**
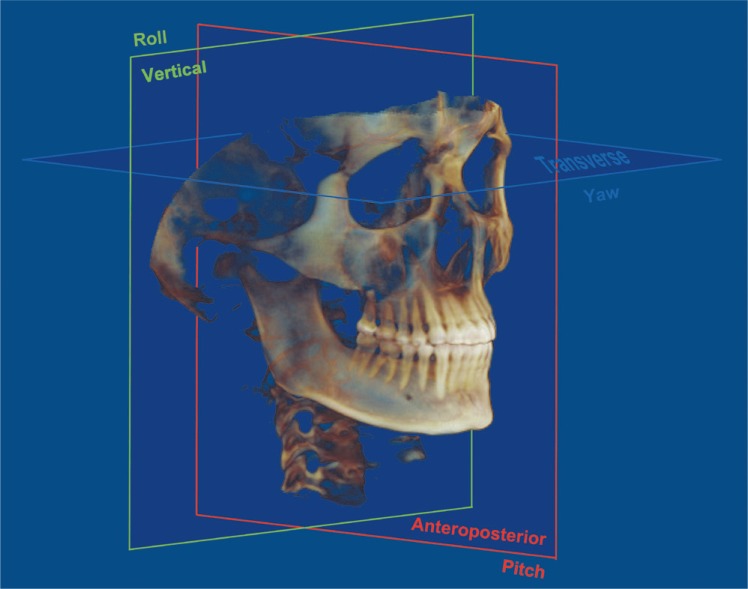
Head orientation based on axial, coronal and sagittal planes.

In order to enable comparison at different times, all images were equally positioned at T_0_ and T_1_. The palatal plane (line connecting posterior nasal spine and anterior nasal spine) and mandibular plane (line tangent to the right inferior border of the mandible) were used as basis for all maxillary and mandibular measurements. Head roll orientation was based on the transverse plane intersecting the right and left frontozygomatic sutures. Head yaw orientation was based on vertical plane tangent to the posterior border of both external acoustic meatus (Fig 1). 

To determine the following maxillary measures, head pitch orientation was based on palatal plane horizontally oriented: (1) intermolar distance (MD), linear distance between right and left tips of first permanent molars mesio-palatal cusps; (2) vertical displacement of molars (VDM), vertical distance between the tips of first permanent molars mesio-palatal cusps and the palatal plane; (3) interpremolar distance (PMD), linear distance between right and left tips of first premolars palatal cusps; (4) vertical displacement of premolars (VDPM), vertical distance between the tips of first premolars palatal cusps and the palatal plane; (5) posterior maxillary cross-sectional area (PMA), obtained at first molars region. Coronal image slice showing the tips of mesio-palatal cusps was used. The area was delimited by a line connecting right and left palatal alveolar crests contouring the palatal vault; (6) anterior maxillary cross-sectional area (AMA), the region of first premolars, the same method described for PMA was used.

Subsequently, for mandibular measures, head pitch orientation was based on mandibular plane horizontally oriented: (7) intermolar distance (MD), linear distance between right and left tips of first permanent molars mesio-buccal cusps; (8) vertical displacement of molars (VDM), vertical distance between the tips of first permanent molars mesio-buccal cusps and the lower mandibular border; (9) interpremolar distance (PMD), the linear distance between right and left tips of first premolars buccal cusps; (10) vertical displacement of premolars (VDPM), vertical distance between the tips of first premolars buccal cusps and the lower mandibular border; (11) posterior mandibular cross-sectional area (PMnA), obtained at the region of first molars. Coronal section showing the tips of mesio-buccal cusps was used. The area was delimited by a line connecting right and left lingual alveolar crests and contouring the lingual alveolar bones. However, there is no lower anatomic limit for the cross-sectional areas of the mandible. Thus, a straight line connecting the lowest points located at the mandibular border delimited these areas; (12) anterior mandibular cross-sectional area (AMnA), the region of first premolars, the same method described for PMnA was used to obtain this measure; (13) mandibular occlusal area (MnOA), using an axial image slice, this area was calculated connecting all lower dental landmarks; (14) mandibular occlusal contour (MnOC), linear connection of all mandibular dental landmarks, using the same axial image slice described for MnOA; (15) incisor mandibular plane angle (IMPA), the angle formed by long axis of the right mandibular central incisor and the mandibular plane (Figs 3A and B). 

**Figure 2:**
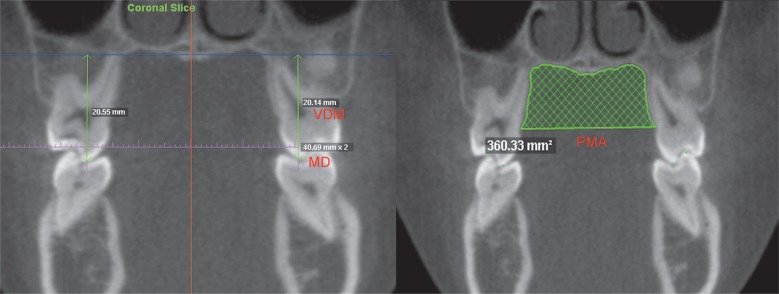
Maxillary measures: (MD) intermolar distance; (VDM) vertical displacement of molars and (PMA) posterior maxillary cross-sectional area. The same measures were performed for premolars.

**Figure 3:**
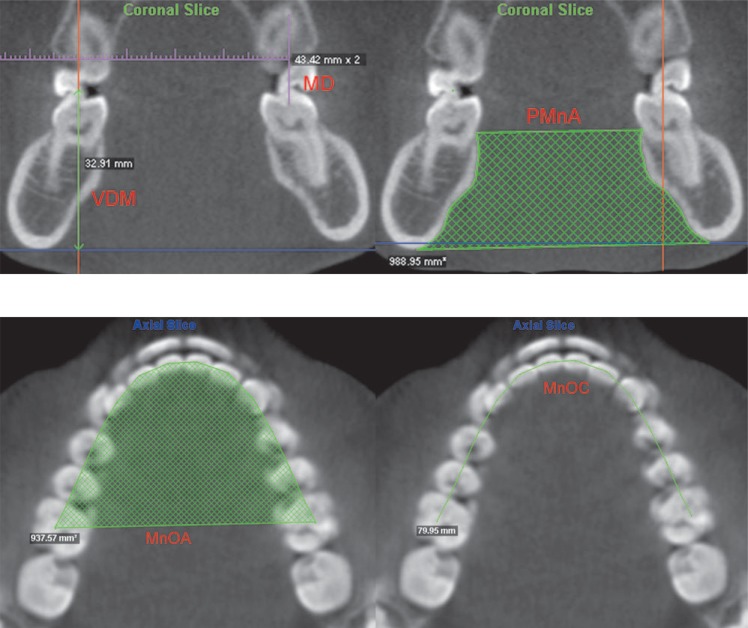
A) Mandibular measures: (MD) intermolar distance; (VDM) vertical displacement of molars and (PMnA) posterior mandibular cross-sectional area. The same measures were performed for premolars. B) Mandibular occlusal area (MnOA) and mandibular occlusal contour (MnOC).

All measures were compared at T_0_ and T_1_. In order to determine reliability and reproducibility, the same examiner used the same protocol to measure three patients, three times with a one-week interval in between. Intraclass correlation coefficient (ICC) was used to determine measurement consistency. Unpaired Student's t-test was used to compare the differences between both expanders (Haas and Hyrax) while paired Student's t-test was used to compare the results at T_0_ and T_1_. Significance level was set at 5%.

## RESULTS

A high ICC value of 0.963 was found, thereby indicating great measurement precision and reliability. Comparison between Group I and Group II at T_0_ and T_1_ is shown in Table 1. No statistically significant differences were found when comparing the main effects of Haas and Hyrax expanders. Thus, the rest of the analysis considered the total sample of 31 individuals.

**Table 1:** Comparison of the main measures of Group I and Group II. Mean values, standard deviations and significances at T0 and T1.

		T_1_			T_0_		
		Group I	Group II		Group I	Group II	
		Mean ± SD	Mean ± SD	Sig. P value	Mean ± SD	Mean ± SD	Sig. P value
Mandible	MD^1^	45.32 ± 2.99	44.45 ± 2.87	0.33	46.86 ± 3.13	46.46 ± 2.98	0.19
	PMnA^2^	931.47 ± 97.94	919.75 ± 97.36	0.26	971.31 ± 142.95	961.12 ± 87.70	0.22
	PMD^1^	33.27 ± 1.67	33.65 ± 3.45	0.12	35.72 ± 3.12	34.71 ± 2.72	0.11
	AMnA^2^	746.66 ± 123.80	738.81 ± 117.25	0.30	787.05 ± 145.28	776.59 ± 100.33	0.27
	MnOA^2^	1006.42 ± 83.95	991.37 ± 62.18	0.29	1045.94 ± 103.80	1033.76 ± 91.21	0.37
	MnOC^1^	83.62 ± 4.36	82.92 ± 4.15	0.65	84.79 ± 4.32	84.52 ± 4.28	0.86
	IMPA^3^	92.88 ± 4.72	91.97 ± 5.77	0.87	93.43 ± 4.55	94.38 ± 5.40	0.75
Maxilla	MD^1^	39.80 ± 2.89	39.79 ± 1.99	0.61	45.41 ± 2.66	45.13 ± 2.10	0.58
	PMA^2^	344.14 ± 57.91	341.12 ± 46.14	0.13	400.14 ± 58.86	397.74 ± 53.35	0.12
	PMD^1^	29.61 ± 2.43	28.95 ± 2.84	0.16	34.91 ± 2.40	35.30 ± 2.65	0.17
	AMA^2^	221.77 ± 59.11	223.39 ± 41.86	0.63	267.13 ± 63.49	265.82 ± 67.82	0.69

S.D. - standard deviation. Sig. - significance P= 0.05. 1 - values in millimeters. 2 - values in square millimeters. 3- values in degrees.

Maxillary intermolar and interpremolar distances increased 5.5 mm and 5.57 mm, respectively. Similarly, mandibular intermolar and interpremolar distances increased 1.74 mm and 1.7 mm. Although these variations were smaller for lower teeth, they were statistically significant. All first molars and first premolars underwent extrusion movements with average values of 0.15 mm (upper teeth) and 0.78 mm (lower teeth). However, these movements were statistically significant only for mandibular teeth.

All areas augmented significantly. PMA and AMA increased 56.18 mm^2^ and 44.32 mm^2^, respectively. The values for the corresponding lower areas were 40.32 mm^2^ and 39.91 mm^2^. MnOA and MnOC increased 43.99 mm^2^ and 1.35 mm. On average, mandibular incisors proclined 1.23^o^. The mean values, standard deviations, differences and significances for all maxillary and mandibular measures are shown in Tables 2 and 3.

**Table 2:** Mean values, standard deviations, differences and significances for all maxillary measures at T0 and T1.

		T_0_	T_1_	Difference (T_1_-T_0_)	Sig. P value
		Mean ± SD	Mean ± SD	Mean ± SD	
Upper molars	MD^1^	39.79 ± 2.51	45.29 ± 2.41	5.5 ± 1.46	0.00
	MVD*^1^	21.13 ± 2.18	21.26 ± 2.31	0.13 ± 1.15	0.54
	MVD**^1^	21.38 ± 2.06	21.52 ± 2.24	0.14 ± 1.11	0.46
	PMA^2^	342.23 ± 52.53	398.41 ± 56.71	56.18 ± 28.78	0.00
Upper premolars	PMD^1^	29.51 ± 2.61	35.07 ± 3.46	5.57 ± 2.4	0.00
	PMVD*^1^	23.89 ± 2.26	24.02 ± 2.46	0.13 ± 0.98	0.48
	PMVD**^1^	23.83 ± 2.38	24.04 ± 2.33	0.21 ± 0.91	0.14
	AMA^2^	222.5 ± 51.82	266.81 ± 64.22	44.22 ± 39.47	0.00

S.D. = standard deviation. Sig. = significance P = 0.05

* right side

** left side. 1 - values in millimeters. 2 - values in square millimeters.

**Table 3:** Mean values, standard deviations, differences and significances for all mandibular measures at T0 and T1.

		T_0_	T_1_	Difference (T_1_-T_0_)	Sig. P value
		Mean ± SD	Mean ± SD	Mean ± SD	
Lower molars	MD^1^	44.96 ± 2..92	46.7 ± 3.02	1.74 ± 1.29	0.00
	MVD*^1^	31.50 ± 3.67	32.19 ± 3.29	0.69 ± 0.81	0.00
	MVD**^1^	31.52 ± 3.21	32.35 ± 3.13	0.83 ± 0.92	0.00
	PMnA^2^	925.94 ± 108.64	966.26 ± 123.65	40.32 ± 57.49	0.00
Lower premolars	PMD^1^	33.43 ± 2.28	35.13 ± 2.92	1.7 ± 2.69	0.00
	PMVD*^1^	36.22 ± 2.98	36.93 ± 3.22	0.71 ± 0.96	0.00
	PMVD**^1^	36.07 ± 3.1	36.99 ± 3.12	0.92 ± 1.1	0.00
	AMnA^2^	742.6 ± 119.4	782.5 ± 127.97	39.91 ± 59.62	0.00
	MnOA^2^	995.9 ± 76.61	1039.89 ± 99.27	43.99 ± 43.09	0.00
	MnOC^1^	83.33 ± 4.21	84.67 ± 4.23	1.35 ± 1.24	0.00
	IMPA^3^	92.58 ± 7.48	93.81 ± 6.88	1.23 ± 2.41	0.00

S.D. = standard deviation. Sig. = significance P= 0.05

* right side

** left side. 1 - values in millimeters. 2- values in square millimeters. 3- values in degrees.

## DISCUSSION

Intraclass correlation coefficient can be used to determine consistency, reliability and reproducibility of quantitative measurements performed by the same or different observers. ICC values greater than 0.75 indicate excellent reproducibility.^19^^,^ 21 ^,^ 25 The high value of ICC (0.963) found in this study indicates great measurement reliability and precision. Similar values were described by other authors,^17^^,^ 22 ^,^ 23 thereby indicating intraoperator reliability. The high precision of the software measurement tools used, associated with image quality, examiner experience and absence of blurring of anatomic structures^21^ justify these findings.

In the present study, Haas and Hyrax expanders yielded similar results. The effects of RME seems to be similar regardless of the expansion appliance.^4^^,^ 26 The skeletal and dentoalveolar effects produced by these appliances have been the main focus of many studies;^4^^,^ 6 ^,^ 7 ^,^ 11 ^-^ 14 ^,^ 19 ^,^ 26 ^,^ 29 ^,^ 30 ^,^ 32 ^,^ 35 however, when compared to Hyrax, it is assumed that Haas produces more skeletal effects with less teeth inclination.^6^^,^ 7 ^,^ 14 This fact is possibly explained by the presence of the acrylic pad that distributes force through the maxilla, inducing orthopedic modification and remodeling the alveolar processes.^7^^,^ 14 ^,^ 11 ^,^ 29 The main purpose of this study was not simply compare expanders, but investigate intraoral space gains after RME. However, no statistically significant differences were observed when Haas and Hyrax linear measures and cross-sectional space gains were assessed, as shown in Table 1. Nevertheless, these results are based only on quantitative analyses. Qualitative assessments could probably reveal different results based on the superimpositions of Haas and Hyrax cross-sectional images.

Some linear measurements were taken to favor interpretation and understanding of alterations. Intermolar and interpremolar distances increased in both the maxilla and mandible. However, maxilla presented the highest values: 5.5 mm for molars and 5.57 mm for premolars. Expander rigidity assures no flexion or deformation during activation or retention.^31^ Consequently, more significant movement would be expected in anchorage teeth, as stated by other authors.^6^^,^ 14 ^,^ 30 As for the mandible, increases of 1.74 mm and 1.7 mm were observed for intermolar and interpremolar distances. These values are in accordance with those found in other studies^24^^,^ 28 and could be explained by the changes in occlusal contacts after RME. These contacts induce additional loading of the buccal cusps of mandibular teeth, causing expansion and uprighting movements.^10^^,^ 13 ^,^ 15 ^,^ 16 ^,^ 24 Another plausible explanation is associated with oral muscles. During RME, the buccinator is laterally dislocated and the internal presence and function of the tongue could buccally tip posterior teeth, thereby contributing to mandibular interdental distances augmentation.^12^

Maxillary dental extrusion is a common effect related to RME.^5^^,^ 12 ^,^ 13 ^,^ 35 In this study, all first molars and first premolars underwent extrusion movements, as observed in Tables 2 and 3. However, the amount of maxillary teeth extrusion was not statistically significant and expanders rigidity could again explain this effect.^31^ This result is in accordance with Garib et al^7^ who compared 3 groups of patients (Group 1, treated with Haas and Hyrax expanders followed by edgewise therapy; Group 2, treated only with edgewise therapy; Group 3, control group) and found no vertical differences in facial height, maxillary first molars extrusion and overbite. Lione Franchi and Cozza^26^ conducted a systematic review about the effects of RME in growing individuals and concluded that the vertical changes observed after treatment are small and probably transitory. In contrast, mandibular first molars and first premolars presented significantly extrusion. As most patients were in cervical vertebral stage 3 (CVS3),^3^ indicating that active growth was in progress, molars and premolars uprighting movements and dentoalveolar vertical growth could have influenced these results.

A statistically significant increase in maxillary and mandibular cross-sectional areas was observed after RME. With regard to the maxilla, the gain obtained for PMA and AMA was 16.42% and 19.92%, respectively (Table 2). In this study, maxillary separation occurred in all patients, since a gap between maxillary central incisors appeared in all cases. Therefore, these maxillary variations reflect not only alveolar changes resulting from bone remodeling and teeth movement, but also represent orthopedic gains. Phatouros and Goonewardene^33^ found similar results in maxillary cross-sectional areas of patients submitted to RME. In the present study, the authors adopted palatal alveolar crests as reference points to determine the occlusal limits. Since no significant dental extrusion was observed in maxillary molars and premolars, augmentation does not seem to be derived from vertical remodeling of alveolar crests. The type of expander used is another important issue to be considered. As previously stated, some studies assert that the acrylic pad of the tooth-tissue borne expander distributes expanding forces along posterior teeth and the palatal vault, promoting less teeth inclination and more alveolar bone remodeling.^7^^,^ 30 Thus, more space gain would be expected in patients treated with Haas expanders; however, no differences were observed when both appliances were compared. Results are based on quantitative analysis, for this reason, further qualitative assessment including overlap of Haas and Hyrax cross-sectional images could reveal whether space gains are related to specific anatomic regions of the palate or not, thereby promoting better visual understanding of treatment outcomes.^30^ Although space gains were similar for both appliances, differences in shape could reveal the influence of the type of expander used.

As for the mandible, increase was found for posterior (4.35%) and anterior (5.37%) cross-sectional areas, probably as a result of lingual bone remodeling, a consequence of dental uprighting movement, extrusion and vertical growth.^3^^,^ 26 ^,^ 30 ^,^ 37 As previously described, the occlusal limits of mandibular areas were determined by a line connecting the lingual alveolar crests. Since teeth extrusion occurred, vertical bone remodeling of these crests as well as vertical growth could justify these findings. Thus, it seems reasonable to assume that these areas are more reliable to describe alveolar and basal bone changes occurring in the mandible after RME.

On average, MnOA and MnOC increased 43.99 mm^2^ and 1.35 mm. As demonstrated in Table 3, increases in mandibular intermolar and interpremolar distances contributed to yield such results. Other authors confirm uprighting movements of mandibular molars and premolars after RME.^12^^,^ 24 ^,^ 28 Mandibular incisors proclined significantly, as confirmed by IMPA variation, thereby contributing to MnOA and MnOC alterations. However, Tai et al^34^ also found IMPA and intermolar distance increases in untreated patients. Although these changes were not statistically significant, the authors identified a natural tendency towards incisor proclination and molar uprighting. 

**Figure 4:**
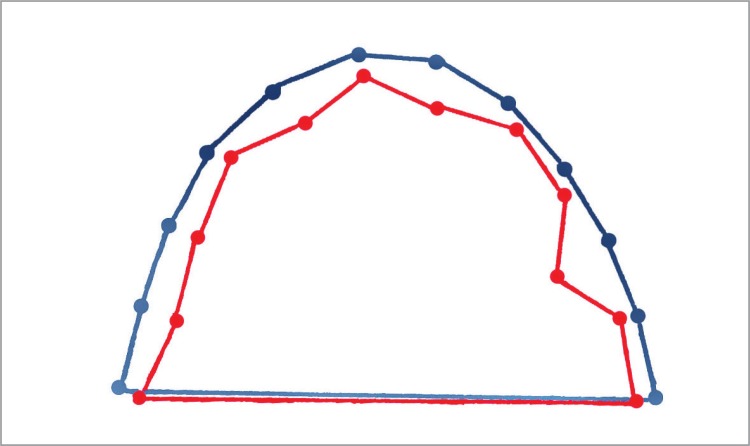
Blue and red landmarks are 1 cm apart. The total contour is 12 cm for both cases. However, differences are evident.

Even though changes in MnOA and MnOC were statistically significant, the clinical relevance of results found for MnOC is questionable. Based on the methodology adopted herein, minor alterations in MnOC could lead to great differences in MnOA, as demonstrated in Figure 4 which shows two examples of mandibular occlusal images represented by blue and red landmarks. These landmarks are 1 cm apart with the total occlusal contour being the same in both examples. Nevertheless, there is great disparity in size, which represents the real difference between them. As a result, the occlusal area seems to represent the occlusal changes in a more reliable manner. It is worth noting that area analysis is not reliable in determining the amount of teeth movement, but it may be a precise tool used to assess intraoral space gains.

The results yielded by the present study are based on two groups treated with RME by means of two different expanders and without a control group. As stated by other authors, the advantage of a control group to compare the results is unquestionable in scientific investigation.^4^^,^ 26 ^,^ 34 However, recent systematic reviews highlighted that most studies based on RME presented some methodological problems including small sample size and absence of a control group.^4^^,^ 26 This is also a limitation of the present study. The methodology adopted herein consists of a longitudinal research based on successive tomographic images. Thus, the control group would be submitted to radiation doses that would not bring any benefits to the individuals, thereby causing potential problems to their health and arising serious questioning in terms of ethical approval. Due to these facts, there were changes during RME and the retention period. The effects of the post-retention period were not observed, once it would implicate in new tomographic images and further radiation doses. Nevertheless, these effects are important and help us understand the stability of this kind of treatment and should be explored in future studies.

## CONCLUSION

Based on the studied sample and on the methodology adopted herein it is reasonable to conclude:

» Maxillary and mandibular cross-sectional areas increased significantly after RME. Mandibular occlusal area also increased.
» No statistically significant differences were found when comparing the effects of Haas and Hyrax expanders.
» Maxillary and mandibular intermolar and interpremolar distances increased after RME.
» Cross-sectional and occlusal analyses seem to be alternative methods to assess intraoral changes after RME.
» Studies in different populations with similar methodology and the presence of a control group would be important to confirm the present results.
